# Use of Real-World FHIR Data Combined with Context-Sensitive Decision Modeling to Guide Sentinel Biopsy in Melanoma

**DOI:** 10.3390/jcm13113353

**Published:** 2024-06-06

**Authors:** Catharina Lena Beckmann, Georg Lodde, Jessica Swoboda, Elisabeth Livingstone, Britta Böckmann

**Affiliations:** 1 Department of Computer Science, University of Applied Sciences and Arts Dortmund (FH Dortmund), 44227 Dortmund, Germany; 2 Department of Dermatology, Venereology and Allergology, University Hospital Essen, 45147 Essen, Germany; 3 Institute for Artificial Intelligence in Medicine, University Hospital Essen, Girardetstraße 2, 45131 Essen, Germany; jessica.swoboda@uk-essen.de

**Keywords:** standard operating procedures, clinical pathways, computer-interpretable clinical guidelines, BPMN, HL7 FHIR, clinical decision-making, patient-specific modeling, malignant melanoma, point of care, real-world data

## Abstract

**Background**: To support clinical decision-making at the point of care, the “best next step” based on Standard Operating Procedures (SOPs) and actual accurate patient data must be provided. To do this, textual SOPs have to be transformed into operable clinical algorithms and linked to the data of the patient being treated. For this linkage, we need to know exactly which data are needed by clinicians at a certain decision point and whether these data are available. These data might be identical to the data used within the SOP or might integrate a broader view. To address these concerns, we examined if the data used by the SOP is also complete from the point of view of physicians for contextual decision-making. **Methods**: We selected a cohort of 67 patients with stage III melanoma who had undergone adjuvant treatment and mainly had an indication for a sentinel biopsy. First, we performed a step-by-step simulation of the patient treatment along our clinical algorithm, which is based on a hospital-specific SOP, to validate the algorithm with the given Fast Healthcare Interoperability Resources (FHIR)-based data of our cohort. Second, we presented three different decision situations within our algorithm to 10 dermatooncologists, focusing on the concrete patient data used at this decision point. The results were conducted, analyzed, and compared with those of the pure algorithmic simulation. **Results**: The treatment paths of patients with melanoma could be retrospectively simulated along the clinical algorithm using data from the patients’ electronic health records. The subsequent evaluation by dermatooncologists showed that the data used at the three decision points had a completeness between 84.6% and 100.0% compared with the data used by the SOP. At one decision point, data on “patient age (at primary diagnosis)” and “date of first diagnosis” were missing. **Conclusions**: The data needed for our decision points are available in the FHIR-based dataset. Furthermore, the data used at decision points by the SOP and hence the clinical algorithm are nearly complete compared with the data required by physicians in clinical practice. This is an important precondition for further research focusing on presenting decision points within a treatment process integrated with the patient data needed.

## 1. Introduction

### 1.1. Background and Significance

Clinical Decision Support Systems (CDSSs) have a high potential to advance clinical decision-making in oncology [1,2] and to provide relevant information at the point of care [3]. For textual guideline-compliant knowledge, the mapping to clinical practice has already been achieved by using Standard Operating Procedures (SOPs) [4]. However, more investigation is needed to evaluate the direct effect of SOPs on patients [5], including the timely provision of accurate patient-specific data [6] for clinical decision-making. For representing a sequence of decisions in cancer guidelines, guideline-based algorithms [7] provide step-by-step decision criteria for any given situation [8]. A step-by-step simulation of patient data along a computer-interpretable SOP, represented as a clinical algorithm, could provide new insights into the completeness of data required for patient-specific clinical decision-making processes. It would also reveal the impact of CDSS on oncology care [9]. To ensure decision-making is patient-specific, the patient’s clinical history and personalized treatment must be considered [10]. This can be achieved using data from time-stamped electronic health records (EHR) at decision points [6] together with appropriate technical standards.

New healthcare interoperability standards have had a significant impact on clinical decision support [11]. The Health Level Seven (HL7) Fast Healthcare Interoperability Resources (FHIR) standard has received widespread attention in the healthcare sector [12] and is the most extensively studied standard in the field of clinical decision support [11] for the representation of patient data. To combine FHIR data elements with clinical algorithms, modeling syntaxes such as Business Process Model and Notation (BPMN) [13] are used to define processes of clinical decision support. Research on the use of BPMN to redesign and optimize clinical processes in the healthcare sector is ongoing [13]. However, a major drawback is the lack of a direct link among BPMN models, their execution, and patient data in EHRs.

Workflows are implemented in personalized oncology to support complex decision-making [14]. As outlined by Weske, simulation techniques are potentially useful in validation purposes, whereas step-by-step simulations are applied to investigate the quality of the logical execution of activities and to determine whether the process exhibits the intended behavior [15]. Simulation processes as a whole generally involve the construction of a simulation model derived from a conceptual model of the business process being analyzed, followed by software implementation [16]. The simulation code is then executed to obtain the desired results [16]. As a standard language for defining business process models, which serve as a basis for the simulation, the notation BPMN prevails [16]. Therefore, we consider the use of a simulation process to be an appropriate method for verification and a valuable basis for providing insights into clinical decision-making at defined decision points.

Related work focuses on integrating BPMN-modeled clinical guidelines into decision support systems, with a service-oriented approach [17]. Other studies have also partially considered the current treatment context of the individual patient [18] but have focused mainly on the technical approach [6]. To the best of our knowledge, evaluating the appropriate usage of complete clinical real-world patient data for decision-making at specific clinical decision points has not yet been studied yet.

Consequently, this drives a research interest in combining a computer-interpretable SOP with corresponding patient EHR data for context-sensitive decision-making and investigating more on if the data used by the algorithm would also be complete and sufficient for physicians. To verify the algorithm, a virtual step-by-step patient walk-through through the standardized treatment pathway (simulation) is provided, accompanying the patient along the modeled clinical algorithm. So, we aim to specify the degree to which the EHR data required and utilized by the clinical algorithm is complete in comparison to the necessary data at the same decision points in clinical practice.

### 1.2. Objectives

First, we verify the algorithm with defined patient datasets from the Smart Hospital Information Platform (SHIP) [19] (p. 294) by simulating them along a modeled clinical algorithm with defined decision points to see if the data that are needed are available. After verification, the data required at the decision points within the algorithm are evaluated by dermatooncologists along the question of how complete it is and where we find discrepancies between the clinical algorithm and clinical practice. Thus, we aim to answer the following two research questions (RQ):

- RQ1 (simulation-related): Are all the data needed at defined decision points available in the EHR, and is it possible to visualize the actual clinical treatment path using this data?
- RQ2 (evaluation-related): Are the data used in the SOP for decision-making complete, and does the SOP accurately define the needed data for a given decision point?

We chose malignant melanoma as our clinical use case because a long-term cohort of 3429 patients with melanoma was available (as of 23 October 2023) in the FHIR-based SHIP data platform. We also had a validated clinical algorithm of a hospital-specific SOP that was previously modeled in BPMN [20] with semantically integrated patient FHIR data [21]. We reduced our cohort to those patients with melanoma who had an indication for a sentinel lymph node excision (SLNE), since there is a clear indication for sentinel biopsy/excision within a German clinical practice guideline and a hospital-specific SOP, guided by a standardized treatment process. SLNE is a diagnostic surgical procedure used to classify the specific stage of a tumor according to the AJCC 8th edition [22] and is currently the subject of intensive clinical research [23,24]. Patients with certain risk factors [20,25] undergo an SLNE and adjuvant therapy is offered for one year if the results are positive.

## 2. Materials and Methods

**Figure 1:**
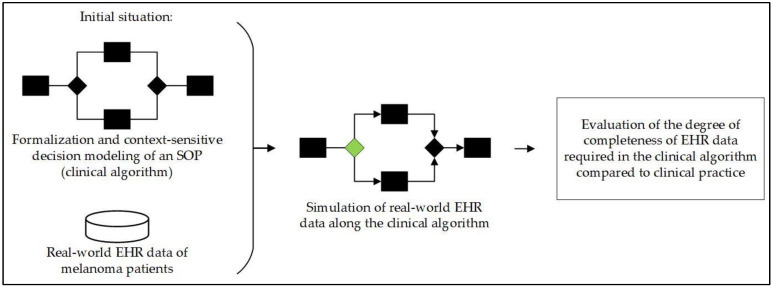
The entire research process, starting with the initial situation, simulation of the real-world EHR data along the clinical algorithm, and evaluation of data completeness at a specific decision point (green). EHR = electronic health records, SOP = standard operating procedure.

The research process is illustrated in Figure 1. This study started with the given clinical algorithm and the real-world EHR data of patients with melanoma. With that data and using the clinical algorithm, a simulation is performed, after which data completeness was evaluated.

### 2.1. Data Collection

**Figure 2:**
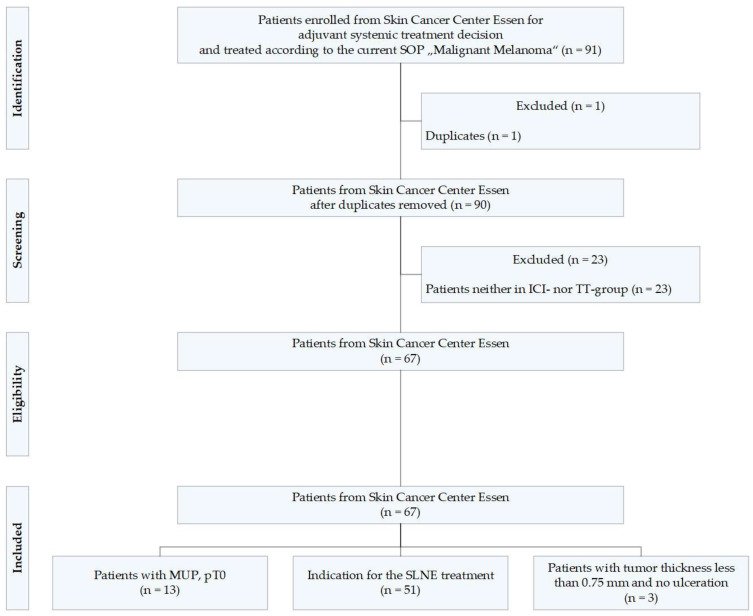
Patient flow chart. ICI = immune checkpoint inhibition; TT = targeted therapy; MUP = melanoma of unknown primary; SLNE = sentinel lymph node excision; SOP = standard operating procedure.

To obtain accurate real-world FHIR data from patients with adjuvant-treated malignant melanoma, we identified melanoma patients who were treated at the Skin Cancer Center Essen, Germany, from a previously conducted multicenter study [26], with corresponding data partly available in SHIP. Our study includes the adjuvant treatment and outcome of patients with stage III melanoma under real-world conditions, as well as patients with melanoma of unknown primary (MUP). A total of 91 patients were enrolled at the Skin Cancer Center Essen, Germany, for adjuvant systemic treatment and were treated according to the SOP Malignes Melanom [Malignant Melanoma] [27]. Duplicate patient datasets and datasets of patients who received neither immune checkpoint inhibition nor targeted therapy were excluded to match the inclusion criteria from our previous study, on which this work builds. The patient flow chart (Figure 2) shows that 67 patients remained in our study.

Each of the 67 anonymized patients’ data are represented by an FHIR bundle sourced from the SHIP and stored as a JavaScript Object Notation (JSON) file and contains patient data from various information systems. Table 1 shows the baseline characteristics of our cohort. The cohort was representative in terms of sex, stage, BRAF status, and ulceration status.

In the next step, we acquired the data needed for all decision points in the form of a clinical algorithm as described previously [20]. The resulting clinical algorithm was a guideline-based BPMN model that represented the formalized SOP with a contextual reference to individual patient FHIR data at defined decision points. On this basis, we performed the simulation with the given real-world melanoma patient data along the clinical algorithm to verify it and evaluate the availability and completeness of the required data.

Then, we identified mandatory clinical parameters based on the SOP for real-world patients with an indication for SLNE, including their FHIR queries (Table A1).

**Figure 3:**
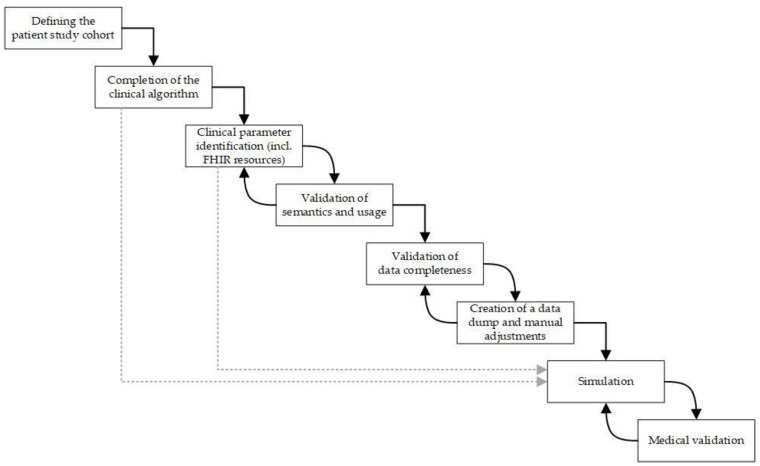
Graphical representation of the pre-processing procedure used for simulation.

Subsequently, we asked a team of experts to perform a medical validation (Figure 3). First, all identified FHIR resources and their fields were validated by a computer scientist of the SHIP team, a dermatooncologist, and a tumor records technician for correct semantics and usage. Second, the completeness of the anonymized patient data was validated using real-world patient data from [26]. An anonymized data dump from SHIP was acquired for manual adjustments resulting from deviations due to unstructured data. Based on the completed clinical algorithm and the validated parameters required for the decision points, the step-by-step simulation was executed along the clinical algorithm using the defined patient cohort. A dermatooncologist evaluated the simulation results by comparing the results with those of guideline-based recommendations for the treatment of stage III melanoma.

### 2.2. Technical Set-Up for Simulation

**Figure 4:**
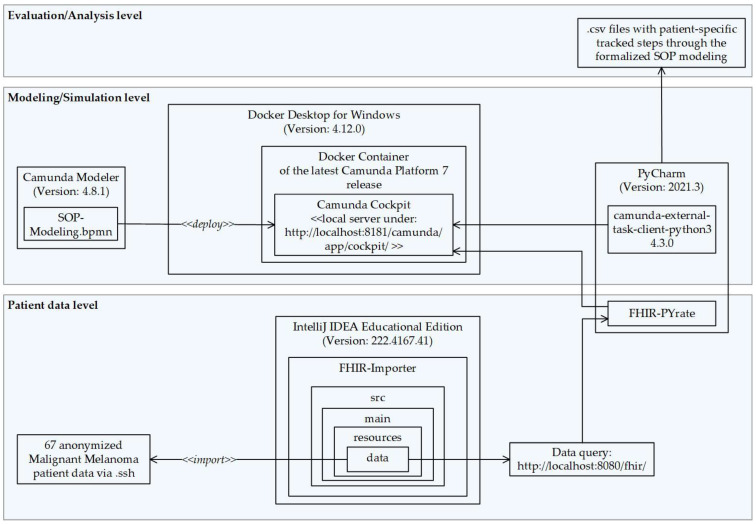
Component diagram of the technical set-up for simulation.

The simulation engine comprised a standard HAPI FHIR server (Apache license version: 2.0, January 2004) using IntelliJ IDEA Educational Edition (version: 222.4167.41); a Camunda Docker container (server version name: Apache Tomcat/9.0.58) and a Camunda Modeler (exporter version: 4.8.1, execution platform: Camunda Platform, execution platform version: 7.15.0), which provided the BPMN model that was deployed on the Camunda Cockpit application (Camunda platform version: 7.18.0). This was all orchestrated by a Python script (using PyCharm version: 2021.3). FHIR data were queried using the FHIR-PYrate package [28]. An overview of the framework is illustrated in Figure 4.

### 2.3. Data Analysis

To analyze the retrospective patient walk-through through the clinical algorithm, each simulation step (BPMN task, gateway, outgoing sequence of the gateway, used parameter to make a clinical decision at a gateway, and end event) was tracked as a table entry and merged into a comma-separated values (CSV) file (see Figure 4). Each tracked file was manually inspected and validated by a dermatooncologist. After validation, the files were combined, and the most relevant characteristics for treatment decisions—such as the distribution of endpoints, the decision points passed, and the applicable data at the individual decision points—were analyzed. Finally, three of the reviewed decision points were selected for evaluation at the point of care.

### 2.4. Evaluation at the Point of Care

We recruited 10 dermatologists (two male, eight female) from the Department of Dermatology of the University Hospital Essen, Germany, by telephone, e-mail, and personal contacts. These participants had different levels of medical experience: two participants had <1 year, one had exactly 1 year, and seven had between 2 and 5 years of experience. However, all the participants were familiar with the hospital-specific processes, the hospital-specific document SOP Malignant Melanoma, and the treatment of melanoma patients. One participant dropped out after evaluating the first decision point (DP1a).

We used the online survey tool Lime Survey, which is hosted on a server at the Department of Computer Science of the University of Applied Sciences and Arts Dortmund, Germany. Participation in the survey was completely anonymous. Each participant answered three personal questions about their sex, experience in dermatology, and experience in dermatooncology, and twelve questions about the decision-making processes along the clinical algorithm in combination with specific patient data; these were divided into the four decision situations: DP1a, DP1b, DP8, and DP16 (the decision situation DP1 was presented twice), each containing three questions. The decision points identified as representative in the previous data analysis were selected for this.

**Figure 5:**
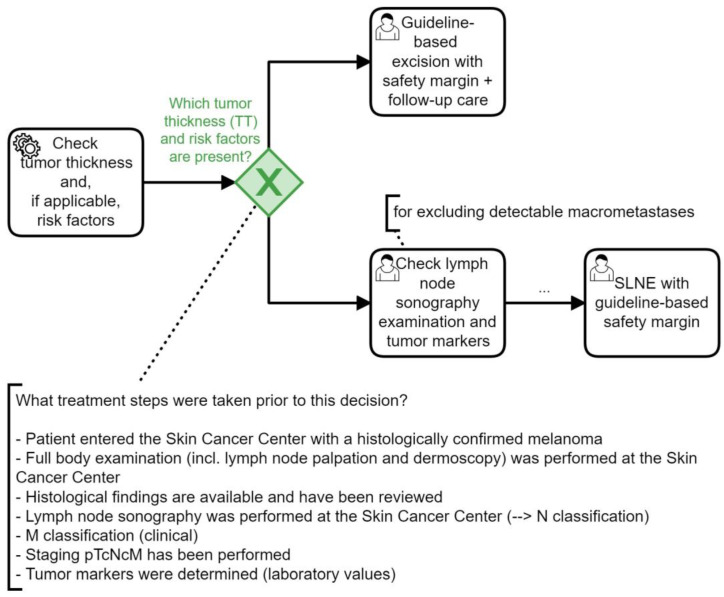
A snippet (translated into English) of the clinical algorithm [20,21] that was presented to physicians to evaluate data needs at the decision point (highlighted in green), here exemplified by decision point DP8 (see Table 2).

In each of the four survey sections, participants were given a snippet from the clinical algorithm for the specific decision point (see Figure 5 for the third decision situation), and the available patient data were presented in a table. We used two representative patient cases from our cohort (see Table 1): a stage IIIC patient for the first decision situation and a stage IIIB patient for the second to fourth survey decision situations.

For the first decision situation, we included missing patient data (tumor thickness) to increase the awareness and understanding of the clinical algorithm. The dermatooncologists were asked to abort the decision if the data were missing, as it is the case in the simulation.

## 3. Results

This study addresses two research questions by examining the availability and completeness of data at decision points. First, we examined the pre-processing, availability, and completeness of EHR data using a simulation of retrospective treatment visualization. Second, the completeness of the required data at certain decision points within the clinical algorithm was evaluated by comparing it to the data available in clinical practice.

### 3.1. Pre-Processing Results

During pre-processing, experts identified 16 clinical features as relevant for decision-making at key decision points to simulate patient data along the clinical algorithm. All clinical parameters could be mapped to FHIR resources and fields in the used FHIR-based SHIP (Table A1) without loss and were thus available. The FHIR queries served as the basis for embedding the patient’s EHR into the simulation. For parameters in the data dump where FHIR queries revealed incomplete data, manual re-editing was performed using data from the multicenter study [26]. Once data completeness was ensured, the simulation proceeded.

### 3.2. Simulation Results

**Figure 6:**
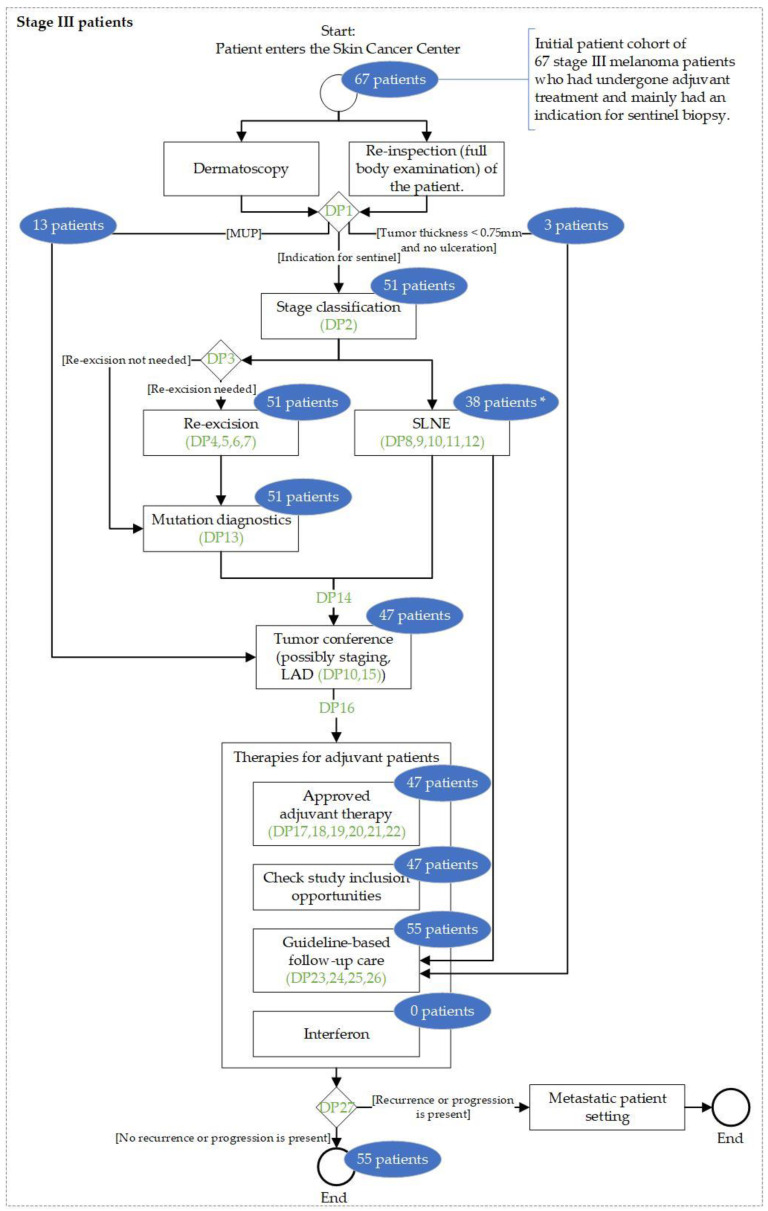
Abstract flowchart of the treatment of melanoma stage III-patients according to the SOP document (from DP1 onwards, a distinction is made among patients with an indication for a sentinel biopsy, MUP patients, and patients who only receive guideline-based follow-up care). The sequence of decision points (green, listed in Table 2) and the proportionate number of the 67 adjuvantly treated patients from our initial cohort who passed through the individual treatment sections during retrospective simulation (blue) are shown. * 12 patients left the SLNE section because of highly individualized treatment in terms of modeling the clinical algorithm, and one patient left because there was no risk constellation to receive an SLNE. DP = decision point, MUP = melanoma of unknown primary, SLNE = sentinel lymph node excision, SOP = standard operating procedure.

**Simulation Characteristics:** The simulation successfully represented the clinical algorithm for all 67 patients, allowing for retrospective analysis. However, twelve patients (see Figure 6, *) could not be fully represented by the clinical algorithm due to the need for highly individualized treatment. This was realized in the clinical algorithm using a preliminary end event. From the clinical algorithm, we identified a total of 27 decision points that a melanoma patient with indication for SLNE might encounter during medical treatment (see Table 2).

**Table 2:** Defined decision points (DP) in the clinical algorithm and the number of patients passing these points during the simulation. The resection status R0 was already specified as an inclusion criterion for the patient cohort at the beginning of the simulation.

ID	Defined Decision Points	Number of Patients Successfully Passed
DP1	What is the patient’s treatment goal in the Skin Cancer Center Essen?	67
DP2	Is the tumor ulcerated AND/OR the tumor thickness > 0.75 mm?	51
DP3	Resection status: In case of R1, complete excision with safety margin; in case of R0, safety margin.	51
DP4	What is the residual classification?	51
DP5	Is there an anatomical, difficult localization (face, acra, etc.) or a non-resectable condition?	51
DP6 *	If the resection status is R1, is an R0 resection status possible?	0
DP7	What is the tumor thickness according to Breslow?	51
DP8	Which tumor thickness and risk factors are present?	51
DP9	Are the lymph node sonography findings unremarkable?	39
DP10 ^1^	Is the patient MRI-compatible?	72
DP11	Is the SLNE status positive?	38
DP12	Is there evidence of metastases?	25
DP13	Is the melanoma stage ≥ IIC?	51
DP14 ^2^	What is the SLNE status?	34
DP15	Is there an indication for LAD?	47
DP16	What steps does the tumor conference decision specify for adjuvant patient treatment?	47
DP17	What does the tumor conference decision specify regarding the indication for adjuvant/systemic therapy?	47
DP18	What is the indication for systemic therapy?	47
DP19	Is a port implantation planned?	28
DP20	Which adjuvant therapy will be used?	28
DP21	Which of the three adjuvant therapies will be used?	19
DP22 *	What does the tumor conference decision specify after the occurrence of side effects or a recurrence?	0
DP23	What is the patient’s tumor stage?	55
DP24	In which follow-up year is the patient?	53
DP25	Is the patient in the sixth follow-up year or higher?	55
DP26 *	Is the patient in the sixth to 10th year of follow-up?	2
DP27	Does the patient have a recurrence or progression/appearance of metastases?	55

* DPs that were not or barely passed are discussed in detail in the main text. ^1^ High number, as the decision was requested twice during the simulation. ^2^ All 13 patients with melanoma of unknown primary continue the process after this DP. DP = decision point; MRI = magnetic resonance imaging; SLNE = sentinel lymph node excision; LAD = lymphadenectomy.

**Review of the Salient Decision Points:** “Passing a decision point” indicates that a patient has met all the data-based requirements, and therefore an outgoing path from a decision point of the algorithm is considered true and the simulation continues accordingly. However, two decision points (DP6 and DP22) were not passed by any patient. The reason for not passing DP6 is a failure to meet the inclusion criteria (R0 resection) of the cohort. Furthermore, this study focused on melanoma patients with an indication for SLNE as a clearly defined question. The multifactorial recommendation for adjuvant therapy at DP22 is based on criteria more complex than SLNE and on specific comorbidities that are not included in the SOP. Consequently, we deliberately excluded this decision point from our simulation and generalized the two questions on patient-specific adjuvant therapy (see Table 2, DP20 and DP21), rather than specifying them further. Additionally, two patients who passed the decision point DP26 were identified as exceptions during medical analysis because of their previous melanoma treatment. All other patients in our cohort were at most in their fifth follow-up year at the reference date for the simulation calculation (26 October 2023).

**Visualization of the Patient Paths:** Figure 6 illustrates an abstract flowchart of the adjuvant melanoma treatment by the clinical algorithm, including the logical sequence of decision points from Table 2. It also highlights in blue (see Figure 6) the frequency of each treatment procedure that the 67 stage III melanoma patients from our initial cohort underwent during retrospective simulation.

For data evaluation, we focused on decision points that used a combination of two or more clinical parameters for decision-making, resulting in the following three decision points:

- DP1: What is the patient’s treatment goal in the Skin Cancer Center Essen?
- DP8: Which tumor thickness and risk factors are present, and will the SLNE be performed?
- DP16: What steps does the tumor conference decision specify for adjuvant patient treatment?

Using these decision points, the completeness of data required for clinical decision-making was evaluated.

### 3.3. Results of the Survey Evaluation in Terms of Completeness of Data and Next Steps after Decision-Making

#### 3.3.1. Evaluation of the Completeness of the Required Data

We examined the completeness of the required data at the decision points in our clinical algorithm with the help of the data required for the same decision points in clinical practice. When assessing the relevance of clinical parameters at the defined decision points, the physicians’ responses were not entirely consistent (Table 3, column “Data Required at Point of Care, Evaluated through Survey”). In addition, one participant only selected one answer per question block, despite instructions allowing multiple answers. The availability of additional data (Table 3, last row in the “Survey responses/Parameter” column for each survey question) was reported by at least one participant at each decision point.

The cut-off range for a clinical parameter considered relevant by the physicians for a specific decision point, as determined by the point-of-care survey (see Table 3, column “Data required at point of care, evaluated through survey”), was categorized into three ranges: less relevant (0–33.3%), moderately relevant (33.4–66.6%), and highly relevant (66.7–100%). In Table 3, the relevance of the parameters was color-coded for a facilitated visual comparison, with highly relevant parameters highlighted in dark green (Table 3).

The comparison results (Table 3, right column) were also color-coded. Dark green indicates a high relevance match between the clinical algorithm (Table 3, fifth column, marked “x”) and the survey responses. Light green indicates a high relevance match where the parameter is implicitly used in the clinical algorithm (Table 3, fifth column, marked “(x)”) and considered highly relevant in the survey. Yellow highlights in the comparison column indicate parameters that were highly relevant in the survey but not mentioned in the clinical algorithm for this decision point, indicating the need for further discussion of these parameters.

For DP1a, four parameters matched (tumor thickness was intentionally not selectable as a survey response but was mentioned by 90% of the physicians as required data.); for DP1b, five parameters matched; for DP8, seven parameters matched, and one highly relevant parameter mismatched; and for DP16, four parameters matched, seven highly relevant parameters matched implicitly, and two highly relevant parameters mismatched (see Table 3). Considering the high relevance range, the comparison column shows data completeness in the clinical algorithm of 100% for DP1a and DP1b, 87.5% for DP8, and 30.8% for DP16. If only these values are considered, data completeness tends to decrease the further the patient has progressed in the clinical algorithm.

However, analysis involving a dermatooncologist suggests that parameters that were used implicitly (Table 3, right column, light green) should also be classified as highly relevant. Although the clinical algorithm does not explicitly list these parameters as highly relevant for DP16, they are encompassed within the “tumor stage” parameter [29], which focuses on pathological stage III melanoma. This justification increased the number of relevant parameters at DP16, thereby raising the data completeness from 30.8% to 84.6%.

The parameters (“Resection status”, “Patient age (at primary diagnosis)”, and “Date of first diagnosis”) whose relevance differed between the clinical algorithm and the survey are highlighted in yellow (see Table 3). The analysis with a dermatooncologist confirmed that the parameter “resection status” was correctly not mentioned in the clinical algorithm at DP8. This parameter was utilized at decision points DP1a and DP1b, as well as at other preceding decision points. It served as a prerequisite for initiating patient treatment and simulation, thus eliminating the need for further consideration at subsequent decision points. However, the parameters “patient age at primary diagnosis” and “date of first diagnosis” at DP16 represented a notable discrepancy between the clinical algorithm and clinical practice.

In summary, the evaluation of data at the three selected decision points (DP1, DP8, and DP16) showed that the clinical algorithm has a minimum completeness rate of 84.6% per decision point. The results also indicated that data must be presented at the specific decision point during treatment and that even if a physician does not require a certain parameter, this parameter must nevertheless be displayed in the dataset for all other physicians. Consequently, ensuring data completeness is essential.

#### 3.3.2. Evaluation of the Next Treatment Steps Based on the Required Data

In addition to evaluating the completeness of the data used at decision points, the selection of subsequent treatment steps based on the required data was also examined. The current decision point and the possible subsequent treatments were presented to the physicians using excerpts from the clinical algorithm (see Figure 5) and a list to mark the appropriate next treatment step. In general, the physicians’ decisions were consistent with the simulation across all four survey categories (Table 4, right column). The entries marked in yellow (Table 4) represent mismatches between the simulation of the clinical algorithm and the survey, indicating where further discussion is needed.

The survey provides details into the first decision situation (DP1a), where a subsequent treatment step could not be determined because of missing data (tumor thickness was missing from the list of available data). In this scenario, 90.0% of the participants selected this option (“Due to a lack of data, the next treatment step cannot (yet) be determined”) indicating the missing data. They also noted tumor thickness was a missing parameter for decision-making in the comment field. One participant did not specify the missing parameter. In each of the two subsequent decision situations (DP1b and DP8), all participants decided identical to the simulation. The last decision situation in the survey concerned the decision point DP16, which defines the final steps for adjuvant patient treatment, similarly as determined by the tumor conference. Here, multiple answers for possible further treatment steps were allowed and were selected by the physicians. The deviation of one participant in the survey on “study inclusion options” can be attributed to the participant who only gave one answer per question block, as previously mentioned. According to a dermatooncologist, the deviation in the option “Guideline-compliant follow-up” was due to a subjective medical understanding. Some dermatooncologists associated this option with “Adjuvant therapy” while a separate mention implied exclusive follow-up care. Since the approval of adjuvant therapy, interferon therapy is no longer part of clinical treatment for stage III melanoma and was therefore correctly classified as not applicable by both physicians and the clinical algorithm. However, this therapy is still included as a therapy option in the SOP.

In summary, as seen in the first part of the evaluation, deviations are mainly noted at DP16, where the decision-making becomes increasingly ambiguous. Otherwise, the simulation and the survey led to the same next treatment steps, which emphasizes the importance of data completeness at each decision point.

## 4. Discussion

### 4.1. Variables

Our study addresses a common limitation found in many CDSSs highlighted by van Baalen et al., wherein treatment suggestions are often generated solely based on diagnosis, without considering unique patient-specific context at key decision points during treatment [30]. In this context, our work incorporates a step-by-step simulation, where the patient-specific decision was made at the decision point itself with the addition of contextual, patient-specific FHIR data. By manually adjusting an anonymized data dump (see Figure 3) and under incorporation of another medical study, we were able to complete our cohort data and use it as basis for simulation. The simulation results (Figure 6) showed that patient treatment can be visualized retrospectively using the available, complete EHR data (cf. RQ1).

### 4.2. Real-World Patient Data

The survey was conducted at the point of care, which is based on the simulation results of real-world patient data along the clinical algorithm. Therefore, we empirically demonstrated the data required for decision-making in clinical practice, as reported previously [8,9]. In contrast to related, rather technical work [6,18], to our knowledge, such an approach exploring data completeness for decision-making has not yet been evaluated with real-world data.

### 4.3. Data Completeness

The evaluation identified the causes of deviations and found a high degree of completeness (between 84.6% and 100.0%) between data required in the SOP and data used by the physicians at the point of care. However, the evaluation revealed that regarding the explicit listing of individual clinical parameters (see Table 3), the SOP could be more specific. Data other than those defined in the SOP were only used for decision-making in few cases, which may be explained by individualized medicine, despite the hospital-specific SOP (cf. RQ2). The frequent occurrence of 88.9% in the responses during the survey (see Table 3) can be explained by the supposed comprehension problem, potentially leading to underestimation of the actual alignment between SOP and clinical practice. It is therefore likely that a higher percentage could have been achieved for some answers if the multiple-choice option were clearer in the survey tool. Conclusively, the matching data between the SOP and survey represent the data a clinician needs at a given decision point, and that completeness of the data in the clinical algorithm is given at a minimum of 84.6%. Thus, it can be assumed that the use of a formalized SOP combined with the provision of complete, accurate real-time patient data at decision points can help optimize clinical decision support.

### 4.4. Limitations

Although our results provide new insights into data evaluation for contextual decision support, our approach considered a very specific medical use case, and our findings may not apply to other tumors or real-time clinical situations, which require further evaluation. Another limitation is that our study examined a small single-center patient cohort (*n* = 67); however, this cohort was representative of our chosen use case, as well as the preceding multicenter study and our research goal. The number of survey participants (*n* = 10) only indicates a preliminary study, consistent with the field of usability testing due to limited participation. Nonetheless, this “end-user testing” method yields around 80% of the necessary insights [31].

### 4.5. Strengths

A strength of our simulation approach is that it builds on the graphical notation BPMN, which is intuitive [32] and so enables rapid adaptation of existing clinical algorithms [20] during interdisciplinary work with clinicians and computer scientists. This is particularly important in the rapidly evolving field of medical science [8]. For example, adjuvant therapy has recently been approved as a treatment for stage IIB/IIC melanoma [23], which could be rapidly integrated into our clinical algorithm. However, the manual effort involved in this work remains a limitation. Firstly, a lot of time was needed for the manual adjustments of those parameters in the anonymized data dump (see Figure 3) for which the FHIR queries returned incomplete data and for the manual modeling of the clinical algorithm. Secondly, the analysis of defined decision points showed that generic modeling via a clinical algorithm can reach its limits when patient treatment becomes increasingly individualized, e.g., the decision on highly specific or off-protocol treatments (see Figure 6, *) or when melanoma patients are given adjuvant therapy (see Table 2, especially DP20 and DP21).

Another strength of our study is the direct extraction of real-world EHR data directly from the FHIR-based data platform, as also addressed in [6], and linking it to a BPMN model. In this way, BPMN models could also be linked directly to an FHIR-based hospital information system, as required in [33], to visualize treatment processes modeled in BPMN using real patient data.

### 4.6. Implications and Future Work

In practical clinical settings, our findings can guide the selection of complete data for specific decision points in melanoma treatment in clinical practice. The applicable section of the model and a list of data required for decision-making can then be visualized in a medical dashboard. Further research could use the patient data to determine the patient’s position within the modeled patient treatment algorithm. Hereby, time stamps of the data should be considered to check if the data would be available in time.

Future research could also simulate a specific time series to enable dynamic reaction to the patient-specific data situation and to automatically determine subsequent treatment steps. This could further enhance the adaptability and efficiency of clinical decision-making processes.

## 5. Conclusions

This study aimed to assess the availability and comprehensiveness of clinically relevant EHR data at critical decision points outlined in an SOP. We conducted a proof of concept by comparing the data requirements stipulated in the SOP against those observed in actual clinical settings. For this purpose, we chose the clinical algorithm of an SOP, validated it by means of retrospective simulation of real-world EHR data, and then evaluated the data required with dermatooncologists. The simulation and evaluation results affirm the availability of data needed in the FHIR-based dataset as well as a high degree of comprehensiveness and accuracy of evidence-based and clinically necessary data at decision points. These are important preconditions to further research focusing on the presentation of decision points within a treatment process integrated with the patient data needed. The results underscore the potential of computerized SOPs as invaluable tools for enhancing clinical decision-making processes.

## Figures and Tables

**Table 1:** Baseline characteristics of patients with malignant melanoma and MUP and with an indication for SLNE.

	All Patients	
	(*n* = 67)	%
Median age at primary diagnosis in years (IQR)	60 (48; 70)	
Sex		
Female	24	35.82
Male	43	64.18
Stage		
IIIA	2	2.99
IIIB	28	41.79
IIIC	35	52.23
IIID	2	2.99
BRAF status		
BRAF mutation	29	43.28
BRAF wildtype	37	55.22
Unknown	1	1.49
T stage of primary (pT)		
T0	13	19.40
T1	5	7.46
T1a	4	5.97
T1b	1	1.49
T2	10	14.92
T2a	6	8.95
T2b	4	5.97
T3	20	29.85
T3a	10	14.92
T3b	10	14.92
T4	19	28.35
T4a	6	8.95
T4b	13	19.40
Ulceration status		
Yes	25	37.31
No	26	38.81
Unknown	16	23.88

IQR = interquartile range; MUP = melanoma of unknown primary; SLNE = sentinel lymph node excision. Adapted from [26].

**Table 3:** Evaluation of data required at specific decision points in our simulation compared with data required by clinicians in the survey (*n* = 10). The relevance of the individual parameters is color-coded as in a heat map: highly relevant (dark green); highly relevant but is only used implicitly in the clinical algorithm (light green); and parameter that needs to be discussed regarding non-listing in the clinical algorithm and is thus a mismatch between the clinical algorithm and the survey (yellow). DP = decision point.

Decision Point	Treatment Context at Decision Point	Survey Question	Survey Responses/Parameter	Data Required in Clinical Algorithm [x = Used, (x) = Implicitly Used]	Data Required at Point of Care, Evaluated through Survey [%]	Match of Data Used in Clinical Algorithm and Survey Responses Classified as Highly Relevant
DP1a	Patient entering the Skin Cancer Center Essen. Dermatoscopy and full body examination have been performed. The next step is to decide on the patient’s treatment goal in the Skin Cancer Center Essen.	What is the patient’s treatment goal in the Skin Cancer Center?	Patient age (at primary diagnosis)	x	70.0%	x
			Sex		10.0%	
			Date of first diagnosis	x	70.0%	x
			Ulceration status	x	80.0%	x
			Resection status	x	80.0%	x
			Additional/other mentioned data		Lymph node sonography findings, Lymph node status, Mitotic rate, (Apparative) Staging, Tumor stage, Tumor thickness	
DP1b	Patient entering the Skin Cancer Center Essen. Dermatoscopy and full body examination have been performed. The next step is to decide on the patient’s treatment goal in the Skin Cancer Center Essen.	What is the patient’s treatment goal in the Skin Cancer Center?	Patient age (at primary diagnosis)	x	66.7%	x
			Sex		11.1%	
			Date of first diagnosis	x	77.8%	x
			Current tumor thickness	x	100%	x
			Ulceration status	x	66.7%	x
			Resection status	x	88.9%	x
			Additional/other mentioned data		General condition/morbidity, Localization of primary tumor, Lymph node sonography findings, Mitotic rate	
DP8	Histology and lymph node sonography have been performed; pTcNcM and tumor markers are available. The next step is to decide whether to perform an SLNE based on tumor thickness and risk factors.	What data do you use to determine the next treatment step?	Patient age (at primary diagnosis)	x	66.7%	x
			Sex		22.2%	
			Date of first diagnosis	x	77.8%	x
			Current tumor thickness	x	88.9%	x
			Ulceration status	x	66.7%	x
			Mitotic rate		55.6%	
			Current melanoma setting		55.6%	
			pT	x	77.8%	x
			Lymph node sonography findings	x	100.0%	x
			Metastasis detection	x	88.9%	x
			Resection status		88.9%	
			Are additional/other data required?		Tumor markers (S100, LDH)	
DP16	SLNE, post-excision, mutation diagnostics, staging, and tumor conference have taken place. The next step is to decide on adjuvant/systemic treatment based on the decision of the tumor conference.	What data do you use to determine the next treatment step?	Patient age (at primary diagnosis)		77.8%	
			Sex		22.2%	
			Date of first diagnosis		77.8%	
			Current tumor thickness	(x)	77.8%	(x)
			Ulceration status	(x)	77.8%	(x)
			Mitotic rate		44.4%	
			Current melanoma setting		44.4%	
			pT	(x)	66.7%	(x)
			Lymph node sonography findings	(x)	88.9%	(x)
			Metastasis detection (clinical)	(x)	88.9%	(x)
			Tumor stage	x	88.9%	x
			Resection status	(x)	77.8%	(x)
			BRAF status	x	100.0%	x
			Metastasis detection (radiological)	(x)	77.8%	(x)
			SLNE status	x	88.9%	x
			Capsular breakthrough	x	88.9%	x
			Are additional/other data required?		Staging, tumor markers (S100, LDH)	

**Table 4:** Evaluation of decisions made at specific decision points by the simulation compared to the survey by clinicians (*n* = 10). The matching (marked by “x” in the right column) and deviations (highlighted in yellow) in the decision-making process between the simulation and the survey are marked accordingly in the table. DP = decision point, MUP = melanoma of unknown primary, SLNE = sentinel lymph node excision.

Decision Situation	Survey/Clinical Algorithm Responses for Further Patient Treatment Steps	Decisions Made by the Simulation (x = Applicable)	Decisions Made by the Survey [*n*, %]	Match between the Decision Made in the Simulation and in the Survey
DP1a: What is the patient’s treatment goal in the Skin Cancer Center? (*only one answer is possible; the decision situation contains missing data*)	The present patient is an MUP patient: connection to the skin tumor center, then staging			x
	Indication for SLNE consultation, preceded by a lymph node ultrasound examination to rule out a locoregional lymph node metastasis		1/10, 10.0%	
	Referral to a dermatologist in private practice			x
	Non-resectable/metastatic setting			x
	Due to a lack of data, the next treatment step cannot (yet) be determined	x	9/10, 90.0%	
DP1b: What is the patient’s treatment goal in the Skin Cancer Center? (*only one answer is possible*)	The present patient is an MUP patient: connection to the skin tumor center, then staging			x
	Indication for SLNE consultation, preceded by a lymph node ultrasound examination to rule out a locoregional lymph node metastasis	x	9/9, 100.0%	x
	Referral to a dermatologist in private practice			x
	Non-resectable/metastatic setting			x
	Because of a lack of data, the next treatment step cannot (yet) be determined			x
DP8: Which tumor thickness and risk factors are present, and will the SLNE be performed? (*only one answer is possible*)	Guideline-based excision with safety margin plus follow-up care			x
	Check lymph node sonography examination and tumor markers; then, perform SLNE with guideline-based safety margin	x	9/9, 100.0%	x
DP16: What steps does the tumor conference decision specify for adjuvant patient treatment? (*multiple answers are possible*)	Adjuvant therapy	x	9/9, 100.0%	x
	Study inclusion options	x	8/9, 88.9%	
	Guideline-compliant follow-up	x	4/9, 44.4%	
	Interferon		0/9, 0.0%	x

## Data Availability

Restrictions apply to the availability of the patient data, which were used under a research agreement for the current work and so are not publicly available.

## Appendix A

**Table A1:** Filter criteria for clinical parameters relevant to the patient simulation process. Each clinical parameter was obtained patient-related, using “specific_parameter_df[specific_parameter_df [fhir:Patient.id] == id_of_the_specific_patient]”.

Clinical Parameter	FHIR Queries to Access the Data in the SHIP [19] Data Platform
Sex	fhir:Patient.gender
Date of primary diagnosis	fhir:Condition.onsetDateTime
Age (at primary diagnosis) = (Date of primary diagnosis − Birth date)	fhir:Condition.onsetDateTime-fhir:Patient.birthDate
Tumor thickness, Breslow	fhir:Observation.where(code.coding.code = ‘B1AAYPBTUDI’).valueQuantity.value
Ulceration	fhir:Observation.where(code.coding.where(code = ‘B1AAYPBULZ’)).valueBoolean
Mitotic rate	fhir:Observation.where(code.coding.where(code = ‘B1AAYPBMITOS’)).valueQuantity.value
pT	fhir:Observation.component.valueCodeableConcept.coding.where(system = ‘https://uk-essen.de/HIS/Cerner/Medico/TumorDocumentation/pTNM/T’).code
Tumor status lymph nodes	fhir:Observation.where(code.coding.code = ‘C0475754’).valueCodeableConcept.coding.code
Metastasis detection	fhir:Observation.where(code.coding.code = ‘C0449918’).valueCodeableConcept.coding.code
SLNE status (Number of affected sentinel lymph nodes)	fhir:Observation.component.where(code.coding.code = ‘B1AAYTNMASNB’).valueQuantity.value)
Residual classification	fhir:Observation.component.valueCodeableConcept.coding.where(system = ‘https://uk-essen.de/HIS/Cerner/Medico/TumorDocumentation/pTNM/LokaleResidualklassifikation’).code
pTNM stage	fhir:Observation.where(identifier.system = ‘https://uk-essen.de/HIS/Cerner/Medico/TumorDocumentation/pTNM’).valueCodeableConcept.coding.display
BRAF status	fhir:Observation.where(code.coding.code = ‘B1AAYPBG1ER’).valueCodeableConcept.coding.display
Overall tumor status	fhir:Observation.where(code.coding.code = ‘C0475752’).valueCodeableConcept.coding.code
Capsular breakthrough	fhir:Observation.where(code.coding.code = ‘B1AAYPBKDB’).valueBoolean
Follow-up year (Reference date for calculation: 26 October 2023	*Query the date of the pTNM tumor stage* (*date_pTNM*): fhir:Observation.where(identifier.system = ‘https://uk-essen.de/HIS/Cerner/Medico/TumorDocumentation/pTNM’).effectiveDateTime *Calculation of the follow-up year:* int(((datetime.datetime.strptime(‘2023-10-26’, ‘%Y-%m-%d’).date())-date_pTNM).days/365.2425)
